# Rate of acute myocardial infarction in patients with troponin-positive chest pain and unobstructed coronary arteries

**DOI:** 10.1186/1532-429X-15-S1-P227

**Published:** 2013-01-30

**Authors:** Lubna Bhatti, Han W Kim, Michele Parker, Rachid Macwar, Raymond J Kim

**Affiliations:** 1 DCMRC, Duke Medical Center, Durham, NC, USA

## Background

Many patients with troponin-positive chest pain are initially diagnosed with acute myocardial infarction (AMI), but are later found to have nonobstructive coronary artery disease (CAD) by x-ray angiography. CMR may be useful in this diagnostically challenging cohort; the presence and/or pattern of myocardial necrosis identified by CMR may allow differentiation between diverse etiologies such as AMI, myocarditis, Takotsubo cardiomyopathy, among others. AMI may occur despite nonobstructive CAD, since plaque rupture with occlusive thrombosis may be followed by recanalization. Nevertheless, the diagnosis of AMI is critical for patient management. Prior CMR studies of this population report a wide range in the rate of AMI (5%-30%), perhaps because existing studies have limited sample size (20-130 patients). The aim of this study was to determine the rate of AMI in a larger patient population with troponin-positive chest pain and unobstructed coronary arteries and to examine clinical characteristics that may be associated with this diagnosis.

## Methods

This single-center prospective study enrolled consecutive patients who presented with troponin-positive chest pain, had obstructive CAD (>50% stenosis) excluded by invasive coronary angiography, and then were referred for CMR. A comprehensive set of clinical characteristics including CAD risk factors and peak troponin levels were collected. Angiographic atherosclerosis severity was categorized as entirely normal (0%), near-normal (1-25% stenosis), and mild atherosclerosis (>25-50% stenosis). Hyperenhancement in a CAD-pattern on delayed-enhancement CMR was used to determine the diagnosis of AMI.

## Results

**Figure 1:**
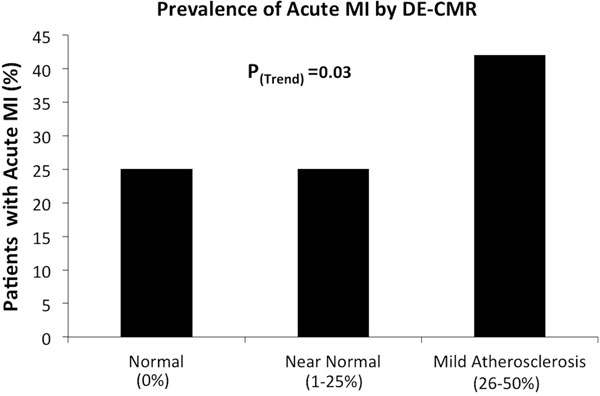
Coronary Artery Stenosis Severity

207 patients were enrolled. Mean age was 55±16 years; 57% were women. The mean number of CAD risk factors was 1.6±1.2. Median peak troponin value was 5.0 x upper limit of normal (IQR 2.6, 12.0). On angiography, 45% had normal coronaries, 28% had near-normal arteries, and 28% had mild atherosclerosis. Table 1 shows the distribution of diagnoses by CMR. The overall rate of AMI was 29.5% (61/207), and a specific etiology was identified in 53% (109/207). No association between the rate of AMI and number of CAD risk factors or peak troponin level was observed (p=0.26 and p=0.17, respectively). Although AMI rate appeared moderately related to the severity of angiographic atherosclerosis (P_trend_=0.03, Figure 1), 25% of patients with entirely normal coronaries also had AMI.

**Table 1:** CMR findings

Diagnosis	N(%)
Myocardial Infarction	61 (29.5%)
Myocarditis	28 (13.5%)
Hypertropic CM	9 (4.3%)
Inflitrative CM (e.g. Amyloid, Sarcoid)	8 (3.9%)
Dilated CM	3 (1.5%)
Nonspecific Necrosis Pattern	4 (1.9%)
Stress CM/Wall motion Abnormaility	54 (26.1%)
Normal CMR	40 (19.3%)

## Conclusions

In over 200 consecutive patients with elevated troponins and nonobstructive CAD, we found nearly one-third had AMI by CMR. Common clinical characteristics such as cardiac risk factors, peak troponin levels, and angiographic atherosclerosis severity have little or no relationship to the diagnosis of AMI.

## Funding

5ROIHL064726-07

